# Comparison of Radiography and Ultrasound for Diagnosis of Rib Fractures in Canine Cadavers †

**DOI:** 10.3390/ani15081087

**Published:** 2025-04-09

**Authors:** Cole Harding, Søren R. Boysen, Cameron G. Knight, Sally L. Sukut, Madison Hillstead, Ashley Finch, Julie Menard

**Affiliations:** 1 Faculty of Veterinary Medicine, University of Calgary, Calgary, AB T2N 1N4, Canada; coleharding@vt.edu (C.H.); srboysen@ucalgary.ca (S.R.B.); cgknight@ucalgary.ca (C.G.K.);; 2 Department of Small Animal Clinical Sciences, Western College of Veterinary Medicine, Saskatoon, SK S7N 5B4, Canada; sally.sukut@usask.ca

**Keywords:** POCUS, greenstick fracture, trauma, diagnostic imaging, necropsy, hemithorax, complete fracture

## Abstract

Human studies suggest point-of-care ultrasound (POCUS) is superior to radiographs for diagnosing rib fractures, but its efficacy in veterinary medicine remains unclear. This study aimed to compare the sensitivity and specificity of POCUS and digital radiographs for detecting rib fractures in canine cadavers, using necropsy as the reference standard. Nine canine cadavers were randomly assigned to either a fracture or control group, with fractures created surgically. Blinded evaluations were performed by an expert and novice sonographer, as well as a board-certified radiologist and a novice radiograph interpreter. Sensitivity and specificity for detecting rib fractures were 83% and 99.74% for ultrasound and 82% and 99.22% for radiographs, with no significant difference between modalities. However, the time required to identify rib fractures varied significantly, with ultrasound taking considerably longer than radiograph interpretation. The expert and novice sonographers required an average of 26 and 64 min, respectively, whereas the radiologist and novice radiograph interpreter took 3 and 10 min. These findings suggest that POCUS and radiographs provide comparable accuracy in detecting rib fractures in canine cadavers. Excluding the time required to obtain radiographs, ultrasound takes longer than radiograph interpretation to identify rib fractures. While POCUS remains a valuable diagnostic tool, its practicality in a clinical setting needs further investigation.

## Simple Summary

Detecting broken ribs in animals is important for diagnosing injuries, but the best way to do this is unclear. This study compared two common imaging methods—X-rays and ultrasound—to see which is better at finding rib fractures in dogs. To test this, we created rib fractures in some dog cadavers while leaving others uninjured. Experts and veterinary students then used ultrasound and X-rays to examine the ribs, and their findings were compared to results from a post-mortem exam. Both methods were very accurate, with little difference in their ability to detect fractures. However, ultrasound took much longer to perform than reviewing X-ray images. These findings suggest that both techniques are reliable, but ultrasound may not always be practical in busy veterinary settings where time is limited. This research helps veterinarians choose the best method for diagnosing rib fractures and may improve care for injured animals in the future.

## 1. Introduction

Rib fractures are a common consequence of blunt thoracic trauma in dogs, particularly following motor vehicle accidents [1,2,3]. Although rib fractures are often self-limiting and rarely life-threatening on their own, their detection is clinically significant, as it alerts veterinarians to potential concurrent intrathoracic injuries and reinforces the need for analgesia.

Rib fractures are frequently associated with more serious injuries, such as pulmonary contusions, pleural hemorrhage and pneumothorax [2,4]. Physical examination alone may not reliably identify rib fractures, except in cases of flail chest, necessitating imaging for accurate diagnosis. Computed tomography (CT) is the preferred imaging modality for detecting rib fractures over radiography [5]. However, both CT and radiography expose patients and personnel to radiation, and require sedation, transport and restraint, all of which can increase risks in patients with respiratory distress. Furthermore, CT imaging may not be accessible for all patients due to financial limitations or limited availability.

In human medicine, similar limitations exist with the ability to diagnose rib fractures based on physical examination. Chest tenderness and deformity have been shown to be very sensitive but not specific for rib fractures, while dyspnea had a low sensitivity and moderate specificity in patients with rib fractures [6]. Similar to veterinary medicine, CT is considered the reference standard diagnostic imaging test in chest trauma patients and is more specific and sensitive than chest radiographs in identifying rib fractures [7]. Although controversial, ultrasound has been used in emergency departments as a bedside modality to diagnose rib fractures, with a meta-analysis study showing ultrasound to be highly sensitive and specific compared to CT, allowing better patient management [8]. In veterinary medicine to date, the use of ultrasound for the detection of rib fractures seems to be limited to horses. In a study in foals, ultrasound identified more rib fractures than radiography [9]. In adult horses, using nuclear scintigraphy as the reference standard for the diagnosis of rib fractures, ultrasound was able to identify a higher number of rib fractures than radiography. In dogs, although ultrasound can be used to diagnose certain fractures (e.g., nasal bones and long bones), there are limited studies demonstrating its use in diagnosing rib fractures [10,11].

The present study aimed to assess and compare the diagnostic accuracy of point-of-care ultrasound (POCUS) and digital radiography in detecting rib fractures in canine cadavers, using necropsy as the reference standard. Specifically, we sought to determine the sensitivity, specificity, positive predictive value and negative predictive value of each imaging modality. We hypothesized that sensitivity, but not specificity, would be different between imaging modalities.

## 2. Materials and Methods

Ethical approval was obtained via the University of Calgary’s Veterinary Sciences Animal Care Committee, protocol AC23-0028. Twelve canine cadavers, euthanized for unknown reasons, were used. Cadavers were included in the study if there was no evidence of prior thoracic trauma, no pre-existing rib fractures, or other apparent rib abnormalities based on screening thoracic radiographs, and the presence of normal skin over the thoracic walls. All cadavers had previously been stored at −20 °C and allowed to fully thaw over 72 h at room temperature. Rib fracture patterns were modeled after a review of the literature [11] and clinical experience on rib fracture distribution in dogs post trauma. For example, dogs struck by motor vehicles more commonly break ribs in clusters, and most often in the caudal one third of the rib cage [5]. Consequently, ribs 1, 2 and 3 were excluded due to their infrequent fracture occurrence and the challenge of creating fractures in these 3 ribs due to their scapular coverage and shape/size.

Fractures were distributed per hemithorax. Each hemithorax was randomly assigned fractures in a four-step process that determined (1) “rib zone”, defined as either ribs 4–7, 8–10, 11–13, or designation as control (no fractures); (2) “number of fractures”, defined as the number of ribs to be broken within the chosen rib zone (1, 2, or all ribs); (3) “exact ribs to be fractured”, being the most cranial, middle, or most caudal rib within the rib zone; and 4) “localization of fracture” on the individual rib (dorsal, middle, or ventral).

**Figure 1:**
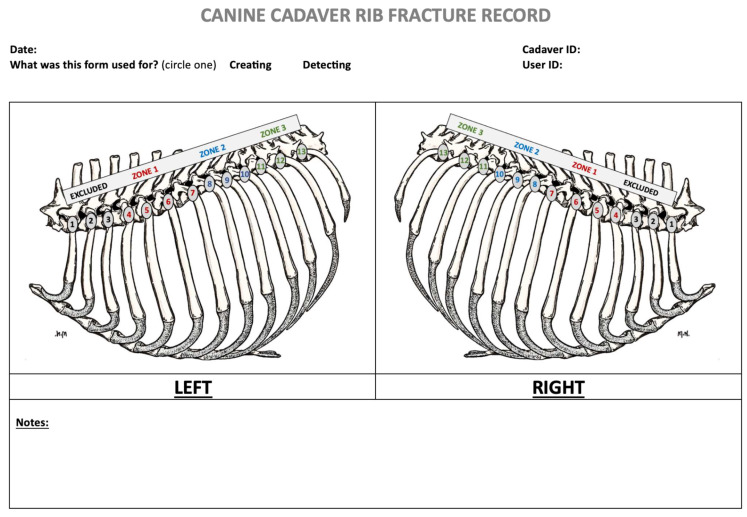
Diagram used to record assignment of rib fractures, creation of rib fractures, sonographic detection, radiographic detection and necropsy detection of rib fractures.

Based on a power analysis with a 0.05 alpha, 80% power and an extrapolated incidence of 23.7% (radiographs) vs. 80.3% (ultrasound) for rib fracture diagnosis from human studies [12], a minimum of 22 rib fractures was required. A total of 32 ribs were assigned fractures, generated using randomization software (https://www.randomizer.org/ accessed on 3 March 2023); of these, there were 18 hemithoraces, with 6 hemithoraces assigned as controls with no rib fractures. Each assigned fracture pattern per cadaver was diagramed for reference (Figure 1).

### 2.1. Rib Fracture Technique

A pilot study was conducted to evaluate the best way to manually break ribs while creating the most realistic radiographic appearance rib fractures induced by blunt trauma. Based on the pilot study, a combined technique utilizing bone cutters and surgical steel chisel and mallet was adopted. Ribs were broken by two investigators not involved in the radiographic or sonographic assessment (JM and AF). The fur on both sides of each cadaver’s thorax was clipped. The ribs were identified by counting backwards from the 13th floating rib. A 2 cm skin and subcutaneous tissue incision using a number 10 scalpel blade was made over the rib at the pre-determined fracture site. Bone cutters were used to score the lateral rib cortex, and a chisel was seated in the resulting superficial cortical defect. A mallet was then used to complete the fracture by striking the chisel handle with blunt force. The fracture was palpated to determine the degree of displacement. Non-displaced fractures were manually displaced ½ to the full thickness of the rib depth using surgical clamps (performed by grasping the circumference of both fracture ends and then physically distracting them lateromedially). Incisions were closed with 3-0 monofilament. In control cadavers, random skin incisions were created and closed over the thoracic wall for blinding purposes.

### 2.2. Sonographic Data Collection

**Figure 2:**
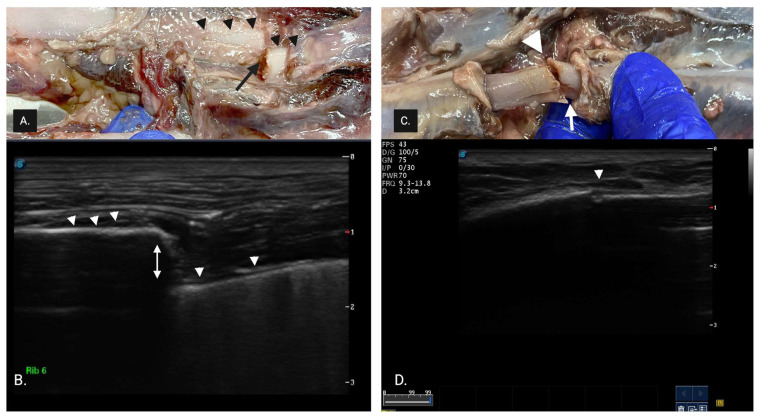
(**A**) Necropsy finding of a complete fracture with displacement. The small black arrowheads represent the superficial cortex of the rib, and the black arrow represents the complete displaced rib fracture. (**B**) Sonographic image of (**A**): complete fracture with discontinuity in the superficial cortical alignment shown by the white arrowhead, and a break between the 2 cortices showing displacement shown by the white arrow. (**C**) Complete fracture with minimal displacement (white arrow). (**D**) Sonographic image of (**C**). The white arrowhead shows a discontinuity in the hyperechoic cortical rib margin.

All cadavers were scanned by an expert sonographer (>20 years POCUS experience) and novice sonographer (final year veterinary student with no clinical sonographic experience). During the pilot study, sonographers familiarized themselves with the scanning technique and the sonographic appearance of rib fractures. A complete fracture (CF) (Figure 2) was described as having one or more of the following: (1) discontinuity in superficial cortical alignment appearing as a break or step in the hyperechoic rib margin, (2) a linear acoustic edge shadow arising from the fractured rib margin and (3) discontinuity in the hyperechoic cortical rib margin.

**Figure 3:**
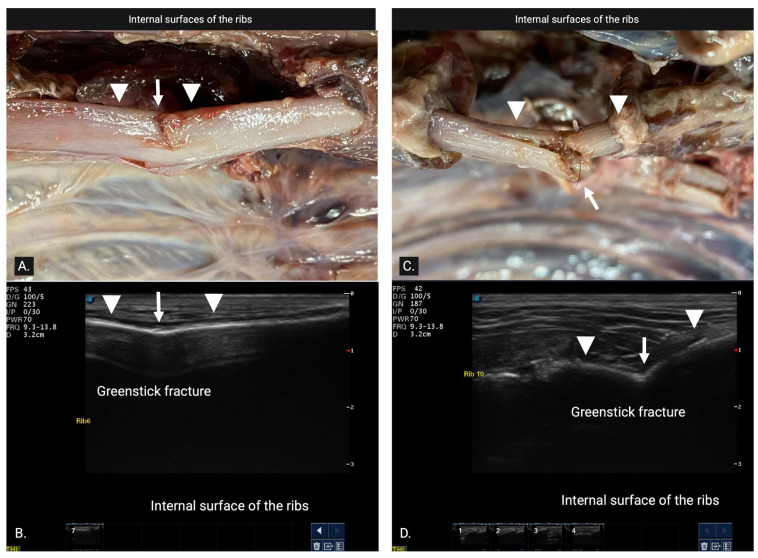
(**A**) External green stick fracture seen on necropsy. The white arrowheads show the superficial surface of the cortex of the rib. The white arrow shows the discontinuity of the superficial surface of the cortex adjacent to muscles of the thoracic wall. (**B**) Ultrasonographic image of (**A**). The white arrowheads show the cortex of the rib. The white arrow shows the small divot in the hyperechoic cortical rib margin with a small loss of cortical integrity, suggesting an external green stick fracture (GSF). It involves only one cortex with superficial cortical disruption limited to bone adjacent to the intercostal muscles. (**C**). Internal GSF seen on necropsy. The white arrowheads show the superficial surface of the cortex of the rib. The white arrow shows the continuity of the cortex adjacent to parietal pleura. (**D**) Ultrasonographic image of (**C**). The white arrowheads show the cortex of the rib. The white arrow shows the pronounced bend in the hyperechoic cortical rib margin without a loss in the cortical integrity in the superficial bone, suggesting an internal GSF. It involves the presence of a smooth superficial cortex despite the significant degree of cortical bending.

A green stick fracture (GSF) (Figure 3) was diagnosed as involving only one cortex and was further categorized into either internal greenstick (superficial cortical disruption limited to bone adjacent to the parietal pleura) or external greenstick (deep surface cortical disruption limited to bone adjacent to the muscles of the thoracic wall) whenever possible.

**Figure 4:**
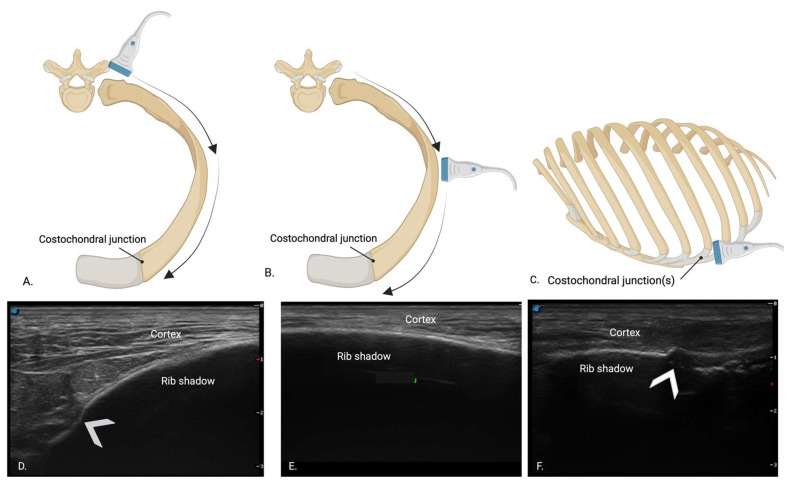
(**A**) Graphic representation of the ultrasound probe over the rib and costovertebral joint. (**B**) Graphic representation of the ultrasound probe over the rib. (**C**) Graphic representation of the ultrasound probe over the rib and costochondral junction. (**D**) Corresponding ultrasonographic image of (**A**). The white arrow represents the costovertebral joint. (**E**) Corresponding ultrasonographic image of (**B**). (**F**) Corresponding ultrasonographic image of (**C**). The white arrow represents the costochondral junction.

All cadavers were scanned using an S9 Sonoscape ultrasound machine (Sonoscape Medical Corp., Guangzhou, China) with a high-frequency (7–12 MHz) linear probe, set to superficial tissue presets using alcohol as a coupling agent. Sonographers were assigned a random scan order for each hemithorax, and the cadavers were kept in lateral recumbency until both novice and expert scans were completed to minimize potential displacement of fractures due to cadaver movement. Sonographers were blinded to one another’s findings and the cadaver specimens. A consistent scanning protocol was used, starting with the most caudal rib (13th rib). The probe was initially positioned perpendicular to the rib to capture the “bat sign”, as previously described [13]. Once the pleural line and rib margin had been identified, the probe was rotated 90° to align over the rib’s long axis. The rib was identified as a bright hyperechoic line with a broad rib shadow beneath it. (Figure 4B,E) The probe was then moved along the rib with slight fanning, rotating, and sweeping motions to maintain alignment over the center of the rib. Using this technique, the rib was scanned dorsally to the point of the costovertebral joint (Figure 4A,D) and ventrally to the costochondral junction to assess for rib fractures (Figure 4C,F). Each sonographer captured still images of fractures and documented fracture types as either GSF or CF on a standardized diagram (Figure 1). Each sonographer assessed all accessible ribs on ultrasound and recorded the time taken to evaluate each hemithorax.

### 2.3. Digital Radiography Data Collection

On the same day as ultrasound scanning, three-view thoracic radiographs (right lateral, left lateral and ventro-dorsal) were obtained (Innovet DXR™, CXDI Control V2.19 software NE, Canon Inc. Irvine, CA, USA, 2012). Care was taken when moving cadavers to minimize any rib fracture displacement. Adjustable DICOM images were then reviewed by an expert board-certified radiologist and a novice radiologist (4th year veterinary student). Time to review each radiograph was recorded for each assessor. Both radiograph reviewers were blinded to the sonographic data, rib fracture placement and pilot study findings. Radiographers documented suspected fractures on a rib cage diagram (Figure 1).

### 2.4. Necropsy

**Figure 5:**
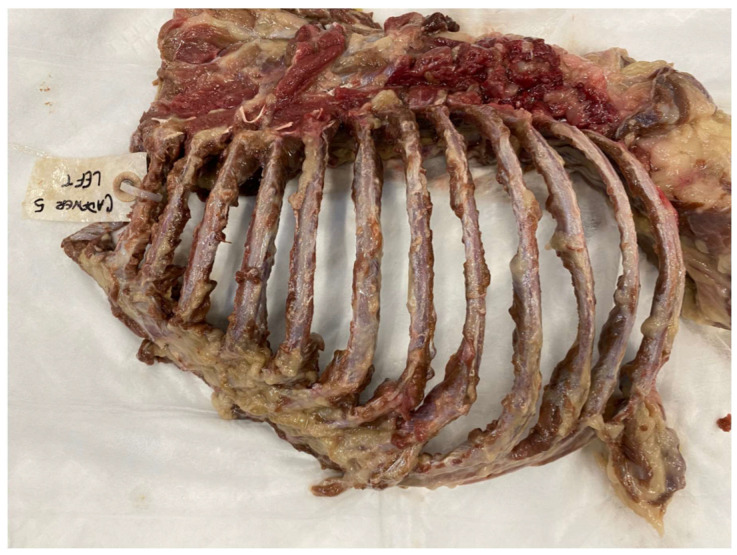
Necropsy evaluation of each individual hemithorax following dissection of the surrounding structures. Necropsy findings were used as the reference standard for comparison.

Following ultrasound and radiography, a necropsy of the thoracic cavity was performed by a board-certified veterinary pathologist in the following way. First, the thoracic limbs were removed by severing all extrinsic muscles at their thoracic attachments. All remaining thoracic skin, fascia and muscle overlying the ribs and external intercostal muscles were removed by blunt dissection, allowing inspection of the superficial rib surfaces. Next, the thorax was isolated from the neck and abdomen by sawing through the seventh cervical vertebra and cutting through soft tissues of the neck at the thoracic inlet, then sawing through the first lumbar vertebra and cutting through the body wall on each side along the thirteenth ribs. Abdominal viscera were severed from their diaphragmatic openings, and the diaphragm itself was removed circumferentially from its attachments to the vertebral column, ribs and sternum. Next, all thoracic viscera was removed bluntly through the diaphragmatic opening, allowing preliminary inspection of the deep surfaces of the ribs. Finally, the thoracic cage was sawn sagittally through the thoracic vertebral column and sternum so that each hemithorax could be fully examined. Each individual rib was thoroughly evaluated only after complete dissection of the surrounding soft tissue attachments and circumferential cortical exposure (Figure 5). Rib fracture locations and types (GSF vs. CF, minimal displacement) identified during the necropsy were recorded on a diagram. Greenstick fractures were further categorized as either internal greenstick or external greenstick.

### 2.5. Statistical Analysis

Results of each hemithorax’s/cadaver’s diagram were manually recorded in a Microsoft Excel document. The D’Agostino and Pearson test was used to test normalcy and the One-way ANOVA/Freidman’s test was used to compare continuous variables between groups with Dunn’s multiple comparison. Differences in time between groups, number of fractures and scan order were assessed with a paired *t*-test, Wilcoxon matched paired rank test and ANOVA. Categorical variables (e.g., fracture vs. intact) were analyzed between participant groups using Chi-square tests. Using necropsy rib fracture identification as the reference standard, sensitivity (Se), specificity (Sp), positive predictive value (PPV) and negative predictive value (NPV) were calculated for each expert and novice ultrasound and radiograph within 3 categories: (1) Total Fractures (TF) combined, (2) CF and (3) GSF. Sensitivity and specificity between expert and novice sonographers’ findings on external GSF vs. internal GSF were compared. A statistical software package (Graph Pad Prism 10 for MacOS, version 10.4.1.532) was used for data analysis with *p* set at ≤0.05.

## 3. Results

### 3.1. Study Population

The dogs were of various breeds and ages and weighed between 13.6 and 28.4 kg. Based on end-of-study necropsy, a total of 50 fractures were ultimately created, with 38 CF and 12 GSF.

### 3.2. Diagnosis of Rib Fractures

Results are shown in Table 1. Whether performed/assessed by novices or experts, ultrasound detection of rib fractures had similar sensitivity and specificity compared to radiographs when necropsy was used as the reference standard, with a *p* value > 0.05 for all combinations.

**Figure 6:**
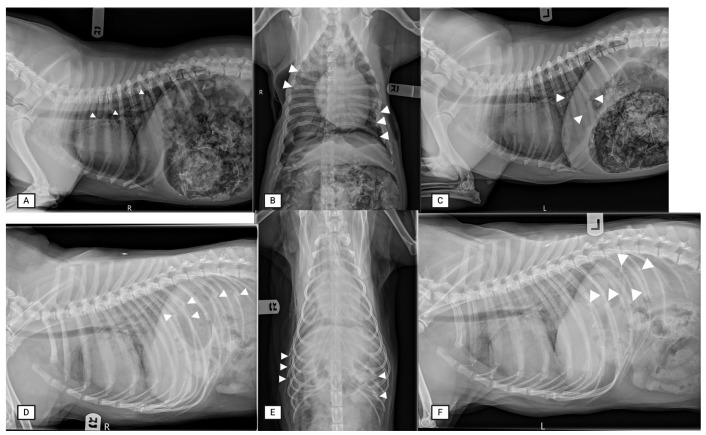
Radiographic evaluation of rib fractures. Three-view thoracic radiographs (right lateral radiographs (**A**,**D**), ventro-dorsal (**B**,**E**) and left lateral radiographs (**C**,**F**) were taken. The points of the white arrowheads show the rib fracture.

Ultrasound allowed for the detection of external greenstick fractures (GSFs) when performed by an expert, with sensitivity and specificity of 100% for both. Internal GSFs were more challenging to identify via the expert sonographer, with a sensitivity and specificity of 50% and 100%, respectively (n = 8). The expert radiologist did not differentiate between greenstick fracture and complete fractures and called all fractures complete. Similarly, both radiography and ultrasound had similar positive and negative predictive values for the identification of complete rib fractures, whether performed by experts or novices (Table 1). False positives were rare, only occurring twice. There were two false positive GSFs, one of which was a CF on necropsy. In both cases, adjacent ribs were positive for fractures. Radiographs detected rib fractures with better accuracy in two cases, both involving a rib with two fractures where only one was detected on POCUS. Lastly, on radiographs, fractures overlying the diaphragmatic crura (zone 3), or those present in the mid dorso-ventral part of the rib, where the convexity of the rib meets the body wall (seen on ventro-dorsal radiograph zone 3), also had occasional missed fractures (Table 2, Figure 6).

### 3.3. Time Taken to Diagnose Rib Fractures

The identification of rib fracture was significantly faster via radiography interpretation compared to ultrasound (Table 3). Within ultrasound, the expert sonographer was significantly faster than the novice. Additionally, there was a learning curve by the expert sonographer, where the scan time was faster for the last 10 hemithoraces compared to the first 10 hemithoraces: Kolmogorov–Smirnov test (*p* = 0.0079). The number of fractures and scan order were assessed for correlation to length of time to scan, with no statistical significance.

## 4. Discussion

The results of this study demonstrate that ultrasound enables a highly sensitive and specific diagnosis of rib fractures in canine cadavers, comparable in diagnostic accuracy to thoracic radiography and corroborated by necropsy findings.

In this study, a complete rib fracture was defined as cortical disruption. Although no universally standardized ultrasonographic criteria exist for diagnosing rib fractures, most studies consider cortical disruption alone as sufficiently sensitive, specific and near-pathognomonic for diagnosis [7,9,14]. However, ultrasound lacks the capacity to differentiate between acute, chronic, or stress fractures [8]. A greenstick fracture was defined as discontinuity in either the superficial or deep cortical alignment and involved only one cortex (either internal for the deep surface or external for the superficial surface). In a related study in foals, no distinction was made between complete and greenstick fractures, although some fractures were categorized as non-displaced, which may correspond to greenstick fractures [9].

Our results align with findings in horses, the only veterinary species with comparable data on rib fractures. In racehorses, ultrasound detected rib fractures in all but one case, as reported by Hall et al. [14]. While nuclear scintigraphy is the reference standard for diagnosing rib fractures in horses by detecting areas of increased bone metabolism for early and sensitive fracture identification, radiographs have limited sensitivity due to overlapping structures and poor contrast. Hall et al. demonstrated that ultrasound was able to confirm fractures following nuclear scintigraphy [14]. Therefore, given its accessibility and cost-effectiveness, ultrasound is the preferred imaging modality for diagnosing rib fractures in adult horses. Similarly, in foals, ultrasound identified fractures in four times as many cases as radiography [9]. In the present study, ultrasound demonstrated similar sensitivity and specificity to radiography for detecting rib fractures in canine cadavers. Consistent with findings in foals [9] and humans [7], false negative and false positive results were also seen on radiographs in our study, likely due to superimposition of viscera and lung pathology reducing radiographic sensitivity and specificity.

Although this was not a primary study aim, results suggest that POCUS may be more sensitive than radiography for detecting greenstick or non-displaced fractures, particularly when performed by experienced sonographers. This increased sensitivity may result from ultrasound’s ability to assess the entire superficial rib surface without superimposition of underlying structures, which can obscure radiographic results. Ultrasound may also detect subtle, incidental bony changes that are not visible on radiographs. Compared with necropsy, the expert sonographer missed only 5 out of 50 fractures, almost all of which were greenstick fractures on the deep rib cortex. This is likely due to challenges in visualizing beyond the hyperechoic external rib surface unless the probe is angled obliquely or fanned under the rib. Due to the small number of GSFs (n = 12 as determined by necropsy), it was not possible to determine the accuracy of internal vs. external GSFs for POCUS or radiographs.

The POCUS technique was less effective at detecting multiple fractures in the same rib, likely due to the linear probe’s wide footprint and difficulty distinguishing closely spaced fractures. Based on power analysis, we initially planned to induce 32 rib fractures at specific locations, identifying ribs by locating the 13th rib and counting backward (cranially). However, in some ribs, more than one fracture was accidentally created in the same rib. In live patients, this would result in a flail chest, allowing rib fracture diagnosis via physical examination. As such, the false negative results are likely less clinically relevant in live patients.

False positives were rare, occurring only twice, and in both cases, adjacent ribs were positive for fractures. Accurately identifying the correct rib number, both for creating and attributing fractures, proved challenging. For both fracture creation and identification, scanning was started by locating the last rib and counting backward (cranially). However, even in cadavers, it was easy to lose count and misattribute a fracture to the incorrect rib by one position.

The novice sonographer demonstrated lower sensitivity than the expert, though this was not statistically significant. This discrepancy is likely due to limited experience in probe manipulation. Previous studies indicate a positive correlation between sonography experience and fracture detection, with medical students learning POCUS detecting long-bone fractures with 15% less sensitivity than experienced radiologists or emergency physicians [15].

In human medicine, numerous studies have shown ultrasound to be superior to radiography and comparable to CT for diagnosing rib fractures in trauma patients. However, in trauma patients, two-view chest radiography remains the first-line imaging modality recommended by both the Canadian Association of Radiologists’ referral guidelines and the American College of Radiology [8]. In most humans studies, ultrasound benefits from patient input, with emergency physicians guiding probe placement to the point of maximal tenderness, improving sensitivity for detecting subtle cortical irregularities [8]. However, in cases of severe trauma where patients cannot communicate, or in those with multiple injuries, a more systematic scanning protocol may need to be performed. This patient profile more closely resembles the canine trauma population, where it is not possible to rely on verbal feedback to localize pain, and where additional thoracic pathologies may also complicate assessment.

In our study, both ultrasound and thoracic radiography exhibited high positive predictive values (PPV) for detecting rib fractures. Given these promising results of ultrasound, studies in live canine patients with thoracic trauma are warranted to determine if these findings extend to emergency department settings. POCUS in canine trauma patients may cause discomfort, especially with rib fractures being present, potentially leading to patient distress and movement, hindering diagnosis of the rib fracture. Studies assessing the feasibility of POCUS for rib fracture identification in live trauma patients are needed. Ultrasound identification of rib fractures may also be helpful following radiographic or CT scan identification, as an aid in targeted pain management, allowing for the precise localization of the fracture and facilitating either blind or ultrasound-guided intercostal nerve blocks [16].

Excluding the time required to obtain radiographs, it took much longer to scan for rib fractures on POCUS than to read radiographs. However, neither method was tested under conditions that would realistically reflect their use on live animals in practice. For example, live dogs would likely require sedation, transport, restraint, repositioning and monitoring to properly obtain radiographs of diagnostic quality [2,17]. Similarly, patients under POCUS may require restraint or analgesia to be amenable to POCUS [18]. As a possible advantage, POCUS offers evaluation at cage-side and is frequently available in many emergency departments during triage [4,17]. Additionally, POCUS can frequently be performed with little support from patient care staff compared to radiography, which may require a team of trained technicians and subsequent radiologist interpretation, which can prolong time to diagnosis [4,17,19]. The literature comparing time between ultrasound and radiography in human practice has found much the same, where ultrasound takes longer than reading radiographs, largely due to the time spent localizing the exact position of the fracture on ultrasound [18,20]. However, most authors still express that the superior sensitivity and accessibility of POCUS outweighs the added time it takes for interpretation [19]. Moreover, some studies found that due to wait times to obtain X-rays, POCUS is actually the faster technique for diagnosing rib fractures, particularly if performed by an experienced sonographer.

Both novice participants required, on average, 2.5 times longer to identify the fractures compared to their expert counterparts. This was expected as efficiency is expected with more experience. Furthermore, the expert sonographer demonstrated a rapid learning curve in the technique, becoming faster over time.

Compared to other diagnostic techniques to identify rib fractures in dogs, such as radiographs or CT [5], POCUS offers a fast, radiation-sparing, portable and affordable option to veterinarians and their clients [2,17,20]. Although radiography and CT are known to have high sensitivity for detecting rib fractures, they are best performed under sedation or anesthesia and require transportation to an imaging suite, where patients in respiratory distress can decompensate [2]. This is in contrast to POCUS, which offers cage-side diagnosis while patients can simultaneously receive supportive care such as oxygen or intravenous therapy [17]. When compared to CT, ultrasound also proves beneficial as each rib can be sequentially scanned parallel to its long axis view, a feature inaccessible to sagittal or transverse CT. Moreover, transverse images on CT prevent complete visualization of entire ribs, which angle caudally, limiting the capacity of CT to detect rib fractures [5,12]. Unlike **CT**, ultrasound can also be performed without the negative effects of motion artifact caused by patient respiration [12,21,22]. Lastly, imaging a rib fracture on ultrasound is not a complicated procedure, and can be learned and performed by any emergency clinician with baseline knowledge in ultrasonography, unlike other advanced imaging modalities, which require radiologist expertise [4,22].

We chose to compare ultrasound with radiography, as CT, though increasingly available in veterinary practices, remains costly, not easily accessible in regular practices and requires time and advanced experience for interpretation. In canine trauma cases, particularly where owners have limited resources or in rural settings without access to advanced imaging, ultrasound offers a readily accessible diagnostic alternative. Ultrasound also avoids radiation exposure, which is particularly beneficial for young or pregnant patients. To confirm rib fractures, we conducted necropsies with individual rib dissections, using these results as our reference standard. To our knowledge, no studies have yet compared CT findings with post-mortem thoracic cavity necropsy in dogs.

Our study had several limitations. We used large-breed dog cadavers to assess rib fracture detection using POCUS, as large breeds facilitated the creation of artificial rib fractures. A pilot study tested methods for simulating rib fractures radiographically similar to trauma-induced fractures in dogs. Despite these efforts, the fractures we created may differ in appearance from naturally occurring trauma fractures on both radiographs and ultrasound. Further studies involving live dogs of various sizes and ages would help determine the applicability of our results. Additionally, the cadavers were frozen and thawed before fracture creation, and factors such as autolysis, the absence of respiration, lung inflation and thoracic wall movement may have influenced our findings, particularly for radiographic interpretation. The lack of lung aeration and gas autolysis likely caused significant superimposition with the ribs, making radiographic interpretation more challenging compared to living patients. In live dogs, increased body condition score and the presence of well aerated lungs may impact the accuracy of ultrasound finding. We hope future studies in canine trauma patients will help further establish the diagnostic value of ultrasound for rib fractures, either as a preliminary tool or as an alternative to advanced imaging such as thoracic radiographs or CT.

## 5. Conclusions

In conclusion, rib fractures can be diagnosed using POCUS with equal sensitivity to radiography based on the current cadaver-based research. The identification of rib fracture via POCUS may have applications in clinical cases when dogs may not be transportable to imaging suites, and can be performed with minimal training, although a learning curve appears evident for both the identification of rib fractures and the time required. Further research is needed to assess whether these findings are applicable in live patients in the clinical setting.

## Figures and Tables

**Table 1:** Detection of rib fractures by ultrasound and radiography compared between novice and expert with sensitivity, specificity, negative predictive value and positive predictive value reported. Three types of rib fractures are described: total fractures, complete fractures or greenstick fractures.

	Total Fractures (*n* = 50)		Complete Fractures (*n* = 38)		Greenstick Fractures (*n* = 12)	
Operator	Se. (%)	Sp. (%)	Se. (%)	Sp. (%)	Se. (%)	Sp. (%)
Expert POCUS	90.0	100.0	94.7	99.5	58.3	99.6
Novice POCUS	76.0	99.5	84.2	98.5	25.0	99.6
Expert 3V TXR	82.0	99.0	*	*	*	*
Novice 3V TXR	82.0	99.5	89.5	99.0	25.0	98.6
	**Total Fractures *(n* = 50)**		**Complete Fractures (*n* = 38)**		**Greenstick Fractures (*n* = 12)**	
Operator	PPV (%)	NPV (%)	PPV (%)	NPV (%)	PPV (%)	NPV (%)
Expert POCUS	100.0	97.5	97.3	99.0	87.5	97.8
Novice POCUS	97.4	94.1	91.4	97.0	75.0	96.1
Expert 3V TXR	95.4	95.5	*	*	*	*
Novice 3V TXR	97.6	95.5	94.4	98.0	50.0	96.0

* Data on expert radiologist for severity of fracture (CF vs. GSF) not obtained. POCUS: point of care ultrasound; 3V TXR: three-view thoracic radiographs; Se: sensitivity; Sp: specificity; NPV: negative predictive value; PPV: positive predictive value.

**Table 2:** Detection of rib fractures by radiography compared between novice and expert with necropsy as the reference standard. Sensitivity (Se), specificity (Sp), negative predictive value (NPV), positive predictive value (PPV) and accuracy reported in percentages. Zones 1, 2 and 3 correspond to the zones described in Figure 1.

	Zone 1					Zone 2					Zone 3				
Operator	Se. (%)	Sp. (%)	PPV (%)	NPV (%)	Ac (%)	Se. (%)	Sp. (%)	PPV (%)	NPV (%)	Ac (%)	Se. (%)	Sp. (%)	PPV (%)	NPV (%)	Ac (%)
Expert	80	99.9	92	97	97	95	100	100	97	98	64	95.5	90	89	89
Novice	80	100	100	97	98	95	100	100	97	98	60	98	90	87	88

**Table 3:** Time taken to diagnose rib fractures on POCUS and to interpret radiographs in minutes. For comparison between radiographs and POCUS, the time to scan for both hemithoraxes were added. Parametric data are displayed as mean and standard deviation (±); non-parametric data are displayed as median and interquartile range.

POCUS Expert	POCUS Novice	Radiography Expert	Radiography Novice
26.3 (±7.8) ^a,c,d^	64.8 (±12.7) ^a,c^	3 (3–4) ^b,c^	10.1 (±2.3) ^a,b,d^

Same-value superscript shows a statical difference between the groups. ^a^ Paired *t*-test *p* value < 0.001, ^b^ Wilcoxon matched paired rank test *p* = 0.0078. ^c^. Wilcoxon matched paired rank test *p* = 0.0039 ^d^. Paired *t*-test *p* value = 0.005.

## Data Availability

The original contributions presented in this study are included in the article. Further inquiries can be directed to the corresponding author(s).

## Abbreviations

The following abbreviations are used in this manuscript:

CF	Complete fracture
CT	Computer tomography scanner
GSF	Green stick fracture
NPV	Negative predictive value
POCUS	Point of care ultrasound
PPV	Positive predictive value
TF	Total fractures
TXR	Three-view thoracic radiographs
Se	Sensitivity
Sp	Specificity
